# Impact of Sub-Clinical and Clinical Compression Socks on Postural Stability Tasks among Individuals with Ankle Instability

**DOI:** 10.3390/healthcare10071271

**Published:** 2022-07-08

**Authors:** Hunter Derby, Nathan O. Conner, Amit Talukder, Aaron Griffith, Charles Freeman, Reuben Burch, Jeffrey D. Simpson, Daniel J. Goble, Adam C. Knight, Harish Chander

**Affiliations:** 1 Neuromechanics Laboratory, Department of Kinesiology, Mississippi State University, Starkville, MS 39762, USA; ag2843@msstate.edu (A.G.); aknight@colled.msstate.edu (A.C.K.); hchander@colled.msstate.edu (H.C.); 2 Department of Human Movement Science, Oakland University, Rochester, MI 48309, USA; nconner@oakland.edu (N.O.C.); dgoble@oakland.edu (D.J.G.); 3 Department of Human Sciences, Mississippi State University, Starkville, MS 39762, USA; at2105@msstate.edu (A.T.); cfreeman@humansci.msstate.edu (C.F.); 4 Department of Industrial and Systems Engineering, Mississippi State University, Starkville, MS 39762, USA; burch@ise.msstate.edu; 5 Human Factors & Athlete Engineering, Center for Advanced Vehicular Systems, Mississippi State University, Starkville, MS 39759, USA; 6 Sports Medicine & Neuromechanics Laboratory, Department of Movement Sciences and Health, University of West Florida, Pensacola, FL 32514, USA; jsimpson1@uwf.edu

**Keywords:** postural stability, compression socks, chronic ankle instability, proprioception

## Abstract

Compression socks are used by a very diverse group of individuals and may potentially have a greater impact on physically diminished or impaired individuals as opposed to healthy individuals. The purpose of this study was to compare the effects of sub-clinical (SC) and clinical (CL) compression socks among healthy (CON), copers (COP), and individuals with chronic ankle instability (CAI). Postural stability was evaluated in 20 participants (11 males and 9 females) using Balance Tracking System Balance platform (BTrackS™) during the modified clinical test of sensory integration in balance (mCTSIB) and limits of stability (LOS) tests. Postural sway parameters were analyzed using a mixed model repeated measures analysis of variance 3 (group: CON, COP, and CAI) by 3 (compression condition: BF, SC, and CL) × 4 (balance condition: EO, EC, EOF, and ECF) for mCTSIB and a 3 (group: CON, COP, and CAI) by 3 (compression condition: BF, SC, CL) × 4 (balance condition: FL, BL, BR, FR) for LOS. Results revealed significantly greater postural stability with both SC and CL compression socks when compared to barefoot conditions. However, no significant differences were observed among groups for compression socks grades. Both SC and CL compression socks may be effective in increasing postural stability.

## 1. Introduction

The ability of a person to maintain center of gravity (COG) within the base of support (BOS) is defined as postural stability [1]. Postural stability is achieved by coordinating movements across several joints with the primary goal of stabilizing the center of mass within an individual’s base of support. The sensory information needed to maintain stability is acquired from visual, vestibular, and somatosensory/proprioceptive systems, and alterations to these systems can affect an individual’s postural control and increase fall risk [1,2]. In addition, postural control strategies are also generated in order to stabilize posture [3]. Proprioception is the primary source of sensory feedback utilized in typical postural control situations (about 70 percent). Proprioception is the sensation of joint and body positions and movements [4,5].

Davids et al. (2003) described proprioception as “sensorimotor system noise”, which enhances movement control and joint position perception [6]. Postural stability and optimal joint positioning are critical to functional performance in basic activities of daily living and athletics. In contrast, sub-optimal positions placing the body outside a neutral position, along with instability, often results in pronounced joint misalignment and injurious kinematics. This can cause an unnatural loading on the structure of the musculoskeletal system, impairing motor control and producing a type of articular instability known as functional instability [7,8].

The body is a closed kinetic chain; therefore, ankle instability could result in altered biomechanics throughout the body, increasing the risk of injury, especially for individuals with poor balance [9]. Similarly to the effects of aging on sensory information and motor outputs, a decrease in sensory feedback is observed in individuals with ligamentous injuries, which reveals that neurological feedback mechanisms originating from the articular and musculotendinous structures are crucial for the maintenance of joint stability [9,10]. This further supported Freeman (1965), who explained that alteration in postural control could be due to the deficits in afferent input from mechanorecpetors in ankle ligaments [11]. These deficits can be a result of lateral ankle sprains. As a result of lateral ankle sprains, the mechanical instability of the ankle can occur due to the affected mechanoreceptors, resulting in joint laxity at the ankle [12]. Therefore, the maintenance of ankle stability is crucial in maintaining postural stability [13]. However, despite a lateral ankle sprain, some individuals (copers) may return to their same level of performance over time [14]. Nevertheless, evidence has shown that compression garments may improve ankle stability in individuals with poor proprioception via stimulation of mechanoreceptors from localized and circumferential pressure to the site of instability [15,16].

Compression garments are used in clinical and athletic settings to increase postural stability, potentially impacting physically diminished or impaired individuals more significantly than healthy individuals [17]. Although the exact mechanism behind compression garments’ impact on neuromuscular performance remains unclear, it is believed that compression garments increase postural stability by stabilizing underlying tissue, resulting in increased proprioception and stimulation of cutaneous mechanoreceptors [17]. Furthermore, Nishikawa and Grabiner (1999) hypothesized that wearing compression socks may increase the motor neuron excitability of the Hoffmann Reflex (H-reflex), potentially stimulating various cutaneous mechanoreceptors and motor neurons, and muscle proprioceptors [18]. These mechanoreceptors in the muscles and joints convey information regarding the body’s posture and movements, playing a significant role in proprioception and motor control. Therefore, it is reasonable to say that this increased efficiency in cutaneous stimulation of mechanoreceptors and proprioception could cause an improvement in performance in at-risk individuals that could benefit from compression garments, such as the injured or elderly. This increase in proprioception can be attained by using several different types of compression garments; some more commonly used garments include adhesive tapes and compression socks. However, evidence has revealed that athletic tape does not benefit performance in weight-bearing tasks, such as standing or the support phases of running or walking [19], which could be due to the lack of pressure on the underlying tissues. Therefore, localized compression on the ankle only is believed to provide an increase in joint perception [19].

Very diverse groups of individuals use compression socks including those who have experienced ankle and foot injuries, individuals who experience lower extremity pain, and individuals who participate in sports and fitness with the primary benefit of improved balance and postural stability. Since localized compression effectively improves balance, it is reasonable to assume that greater compression could provide greater proprioceptive feedback in individuals with chronic ankle instability (CAI). Therefore, the purpose of this study was to compare the immediate effects of two different levels of compression socks (clinical: 20–40 mmHg (CL); sub-clinical: <20 mmHg (SC)) compared to barefoot (BF) in three different groups: healthy (CON), individuals with a history of ankle sprain that did not develop chronic ankle instability—copers (COP)—and individuals with chronic ankle instability (CAI). This study specifically aimed to compare differences in postural stability among CON, COP, and CAI groups; compare the difference in postural stability among BF and CL compression socks and SC compression socks; and assess the interactions among groups and compression sock types on postural stability. First, it was hypothesized that the CON group would have greater postural stability than the COP group, followed by the CAI group. The secondary hypothesis was that CL compression socks would elicit greater postural stability than SC compression socks or no socks (barefoot). The third hypothesis was that the CON group wearing the clinical compression socks would elicit greater postural stability and that both COP and CAI groups would benefit from wearing both clinical and sub-clinical compression socks.

## 2. Materials and Methods

### 2.1. Participants

Twenty participants (11 males and 9 females; age: 21.5 ± 2 years; height: 169.63 ± 9.2 cm; weight: 72.13 ± 16.5 kg) both with and without a history of ankle injuries, with no current existing musculoskeletal, visual, vestibular, neurological, and cardiopulmonary disorders, were recruited for this study. Sample size for this study was determined a priori, based on similar past research [20,21,22]. The study was approved by the university’s Institutional Review Board (IRB) under human subject research protocol number IRB-21-089. All participants were university students or employees between 18 and 30 years of age.

In an attempt to perform further validation based on the previous literature, participants completed the Ankle Joint Functional Assessment Tool (AJFAT) to place them into one of the three following categories: control (CON), copers (COP), and chronic ankle instability (CAI) [22,23]. Based on previous research, a score of 22 out of a possible 48 was used to determine which category the participant was placed in [24]. The following specific criteria were met for the CON group: no history of ankle sprain, free from acute lower extremity and head injuries for the previous three months, free from known equilibrium disorders and chronic lower extremity pathologies, and a score of greater than 22 out of a possible 48 on the AJFAT. In order to be defined as a coper, individuals who have previously suffered from ankle sprains must have been free from pain, weakness, and instability in the affected ankle; have resumed all pre-injury activities without limitation for at least 12 months prior to testing; and have a score greater than 22 out of a possible 48 on the AJFAT. In order to qualify as CAI, participants must have had at least one episode of the ankle “giving way” within the past year, have at least one recurrent sprain between three and six months prior to study participation; perceive pain, instability, or weakness in the involved ankle and attribute the cause of those perceptions to their initial ankle injury; failed to return to all pre-injury level of activity; and have a score less than 22 out of a possible 48 on the AJFAT. Based on the criteria mentioned above, participants in the current study included six uninjured controls (CON), eight copers (COP), and six individuals with chronic ankle instability (CAI).

### 2.2. Experimental Design and Procedures

The experimental protocol followed a mixed model repeated measures design consisting of one day of testing preceded by a five-minute familiarization period. During familiarization, following obtaining the informed consent form, a physical activity readiness questionnaire (PARQ) was completed to assess the current physical activity level [25]. In addition, participants’ general anthropometric assessments, including age, height, body mass, and injured foot/ankle, were recorded. The balance testing was assessed using the Balance Tracking System (BTrackS™) Assess Balance Advanced software (Balance Tracking Systems, Inc., San Diego, CA, USA). Participants completed two protocols while barefoot (BF), while wearing sub-clinical compression socks (SC), and while wearing clinical compression socks (CL). Testing protocol began with BF trials, followed by each compression sock condition in a counterbalanced assignment order. Following each sock condition was a 5 min rest period in an attempt to a prevent learning effect. The protocols were as follows.

**Figure 1:**
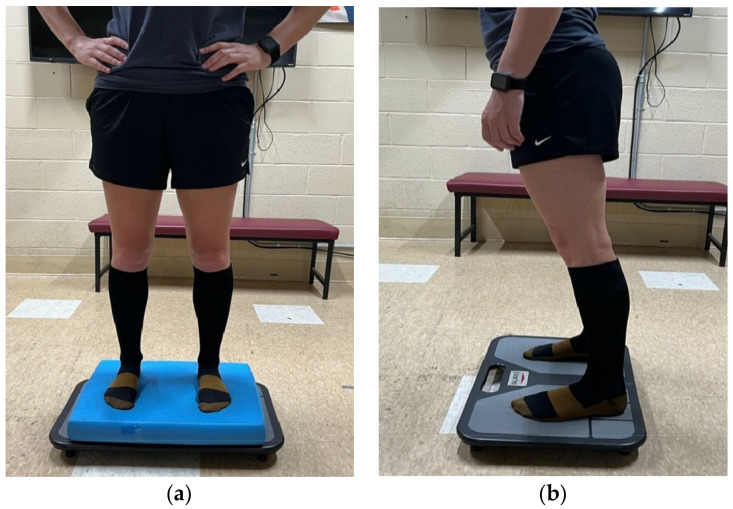
An example of a participant (**a**) performing the foam conditions (unstable surface) on the modified clinical test of sensory integration on balance (mCTSIB) and (**b**) performing the limits of stability (LOS) test on a hard firm surface wearing compression socks.

Modified Clinical Test of Sensory Integration and Balance (mCTSIB): In this assessment, participants were instructed to stand still for four 20s trials on the BTrackS™ balance plate (Balance Tracking Systems, Inc., San Diego, CA, USA) with feet shoulder-width apart and hands placed on the hips. Trials included conditions with both eyes open and eyes closed and on either the stable surface of the plate or on a foam surface placed on the plate. In particular, conditions included eyes open firm surface (EO), eyes closed firm surface (EC), eyes open foam unstable surface (EOF), and eyes closed foam unstable surface (ECF) (Figure 1). Participants completed one trial each condition for a total of four trials that were performed in the same order based on the guidelines of the clinical test as well as the order based on the BTrackS™ software (Balance Tracking Systems, Inc., San Diego, CA, USA). A 5 min rest period was employed following completion of all four trials prior to continuing to the following test.

Limits of stability (LOS): To complete this test, participants stood on the BTrackS™ balance plate (Balance Tracking Systems, Inc., San Diego, CA, USA), with feet shoulder-width apart and arms alongside of the body. Participants were instructed to lean their body in all directions (360 degrees) to create the biggest blue area possible in the middle of the computer screen to the front of them while keeping feet flat on the ground and maintaining balance. Participants were asked to begin by leaning forward as far as possible and then to continue the test by leaning the front left direction, followed by back left, back right, and front right. The area was based on the furthest extent center of pressure (COP) that could be displaced in any given direction and measured in cm^2^. The larger the area created, the greater LOS was (Figure 1). A 5 min rest period was employed following completion of this test prior to continuing to the next sock condition. Completing the balance tests on all three conditions (BF, SC, and CL) marked the end of postural stability testing.

### 2.3. Data Analyses

For mTCSIB, COP Path Lengths (PLs) was derived from the BTrackS™ Balance Plate (Balance Tracking Systems, Inc., San Diego, CA, USA)were calculated by the BTrackS™ Assess Balance software (Balance Tracking Systems, Inc., San Diego, CA, USA). In addition, the front left (FL), back left (BL), front right (FL), back right (BR), and total sway area from the LOS were calculated by the BTrackS Assess Balance software (Balance Tracking Systems, Inc., San Diego, CA, USA). These measures were chosen to provide an overall assessment of the postural stability status directly calculated and derived from BtrackS™ Balance Plate (Balance Tracking Systems, Inc., San Diego, CA, USA).

### 2.4. Statistical Analyses

All balance and postural sway dependent variables were assessed using a mixed model repeated measures analysis of variance (RM-ANOVA) (3 groups (CON, COP, and CAI) (between-subjects)) 3 × 4 (within-subjects) (3 compression condition (BF, SC, and CL) × 4 balance condition (EO, EC, EOF, ECF)) for mCTSIB and a (3 groups (CON, COP, and CAI) (between-subjects)) 3 × 4 (within-subjects) (3 compression condition (BF, SC, and CL) × 4 balance condition (FL, BL, BR, and FR)) for LOS to identify variances between groups and between compression sock grade and type of balance condition. A significant main effect was followed up with post hoc pairwise comparisons with a Bonferroni correction. The significance level was set with an alpha level of 0.05 using JASP statistical software (University of Amsterdam, Nieuwe Achtergracht 129B, Amsterdam, The Netherlands).

## 3. Results

**Figure 2:**
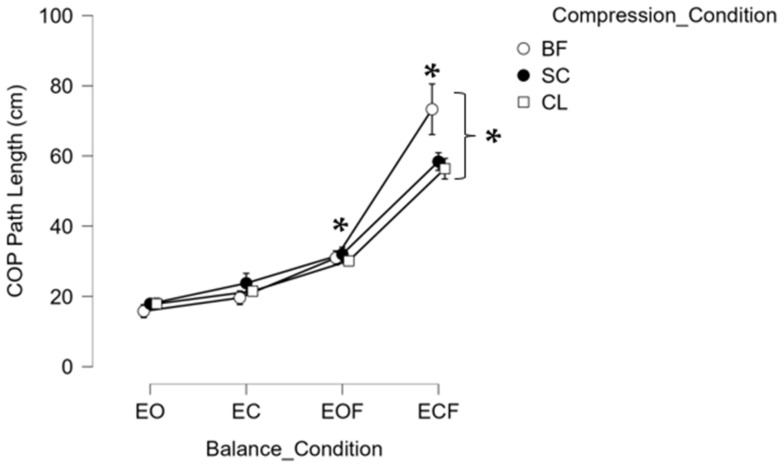
Center of Pressure (COP) path length (cm) during the modified clinical test of sensory integration of balance (mCTSIB) conditions: eyes open (EO), eyes closed (EC), eyes open foam (EOF), and eyes closed foam (ECF) in three compression conditions: barefoot (BF), sub-clinical compression sock (SC), and clinical compression sock (CL). * represents significant differences, and bars represent standard errors.

For mCTSIB, results from RM-ANOVA revealed significant interaction between compression condition (BF, SC, and CL) and balance condition (EO, EC, EOF, and ECF) at F (6, 102) = 4.765; *p* < 0.001, ƞ_p_^2^ = 0.219 and a significant main effect for balance condition (EO, EC, EOF, and ECF) for mCTSIB at F (3, 51) = 95.700; *p* < 0.001, ƞ_p_^2^ = 0.849. Simple effect comparisons of the significant interaction revealed that all compression conditions of BF, SC, and CL had significantly greater COP PL in EOF and ECF compared to EO, significantly greater COP PL in ECF compared to EC, and significantly greater COP PL in ECF compared to EOF. Additionally, BF had significantly greater COP PL in the ECF balance condition compared to both compression sock conditions (SC and CL). Post hoc pairwise comparisons revealed significantly greater COP PL in EOF and ECF compared to EO, significantly greater COP PL in EOF and ECF compared to EC, and significantly greater COP PL in ECF compared to EOF. All significant differences were at *p* < 0.05. However, no significant differences were found between groups (CON, COP, and CAI), and no significant main effect was found across the compression sock conditions (BF, SC, and CL) (Figure 2).

**Figure 3:**
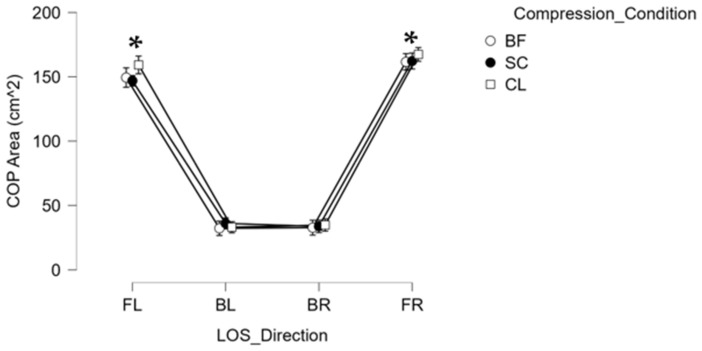
Center of pressure (COP) sway area (cm^2^) during the limits of study (LOS) test in four directions; front left (FL), back left (BL), back right (BR), and front right (FR) in three compression conditions: barefoot (BF), sub-clinical compression sock (SC) and clinical compression sock (CL). * represents significant differences, and bars represent standard errors.

For LOS, results from the RM-ANOVA revealed a significant main effect between LOS directions (FL, BL, BR, and FR) at F (3, 51) = 185.480; *p* < 0.001, ƞ_p_^2^ = 0.916. Post hoc pairwise comparisons revealed significantly greater COP total area for FR and FL compared to BR and BL, indicating that front LOS directions had significantly greater total sway area than backward LOS directions (Figure 3).

## 4. Discussion

This study aimed to determine the effectiveness of sub-clinical and clinical-grade compression socks compared to barefoot conditions during postural stability tasks in individuals with ankle instability compared to healthy controls. Findings from the study supported previous literature that balance testing conditions in which sensory information is absent or altered resulted in decreased postural stability [1]. Regardless of the compression sock condition, COP PL during mCTSIB was significantly greater in EOF and ECF compared to EO, significantly greater in ECF compared to EC, and significantly greater in ECF compared to EOF, suggesting that postural stability is worse when both vision and somatosensory-proprioceptive systems are altered or compromised [1]. A significant compression and balance condition interaction demonstrated that during the more challenging balance condition of ECF, with absent visual feedback and altered somatosensory feedback, the use of both SC and CL compression socks resulted in a significantly lower COP PL compared to the barefoot condition, suggesting better postural stability compared to BF. During LOS, the anterior (forward) directions of FL and FR demonstrated significantly greater COP total sway area compared to the posterior (backward) directions, supporting such directional differences in LOS due to the inverted pendulum model for postural control [26]. However, the lack of significant differences between compression sock types in each direction of the LOS test suggests that compression socks do not impact the LOS in postural control. Finally, while significant differences existed between compression sock conditions compared to barefoot conditions and between balance conditions for both mCTSIB and LOS tests, no significant differences were evident between groups (CON, COP, and CAI). The current investigation included a three-part hypothesis: (1) It was hypothesized that the CON group would have greater postural stability than the COP group, followed by the CAI group, (2) that CL compression socks would elicit greater postural stability than SC compression socks or no socks (barefoot), (3) that the CON group wearing the clinical compression socks would elicit greater postural stability, and that both COP and CAI groups would benefit from wearing both clinical and sub-clinical compression socks. While these findings did not support our hypotheses, the current investigation results support previous research, revealing no significant differences in postural stability between grade compression socks [27].

The mCTSIB on the BTrackS™ Balance Plate (Balance Tracking Systems, Inc., San Diego, CA, USA), along with its four testing conditions of EO, EC, EOF, and ECF, serves to assess the contribution of the visual, somatosensory, and vestibular sensory systems to maintain upright stance and postural stability. Previous literature explains that the CTSIB test may be modified due to lack of differences between healthy and at-risk populations, hence the use of mCTISB [28]. BTrackS Balance Plate (Balance Tracking Systems, Inc., San Diego, CA, USA) has been validated against traditional force platforms, and normative data have been collected on more than 17,000 individuals with ages ranging from 5 to 100 years old [29,30,31]. Significantly better postural stability was evident in conditions when all three sensory systems were available (EO) compared to conditions when the visual feedback was absent (EC and ECF) and when somatosensory feedback was altered (EOF and ECF) [1]. Limits of stability tests have been successfully used to identify fall risk in fall-prone individuals and aid in differentiating fallers from non-fallers [32]. It has also been shown to be useful in assessing functional stability in healthy adults [33]. The limits of stability (LOS) test assesses spatial and temporal postural control measures, including intentional movements of leaning and weight shifts in various directions, maximizing excursions without losing balance and falling. The fear of falling limits ADLs due to constraints in LOS. The current findings support the observation that the anterior directional LOS is significantly greater compared to posterior directional LOS, which can be attributed to the anatomical LOS during static bilateral stance, which, based on the inverted pendulum model suggested by [26], has greater anterior LOS compared to the posterior LOS, especially in the sagittal plane (anterior–posterior direction), with no significant differences between the right and left sides.

Although the mechanism of compression socks’ improvement upon postural control is unclear, it is believed that it could be due to the stimulation of cutaneous mechanoreceptors [17], enhancing proprioceptive feedback. Furthermore, because (gamma) motoneuron activation is primarily influenced by afferent input, decreased proprioceptive input leads to decreased (gamma) motor neuron activation, which, therefore, may lead to decreased joint stiffness and instability. However, Sousa et al. (2017) explained that individuals with CAI as a result of functional ankle instability demonstrate greater proprioceptive deficits than those with mechanical ankle instability [4]. The results of this study provide evidence that compression socks, regardless of the compression grade (SC or CL), significantly improved postural stability, especially when visual feedback was absent and when somatosensory feedback was distorted, suggesting that the compression sock may have improved proprioceptive feedback during such challenging balance testing conditions. The lack of significant differences in other balance testing conditions in the mCTSIB and the LOS can be attributed to the availability of visual feedback and non-distorted somatosensory feedback.

Furthermore, Chang et al. (2022) examined the effects of compression socks on half-marathon runners at different distances [34]. The data revealed no significant differences between compression and non-compression on proprioception until 21 km, implying compression socks have a greater effect with time [34]. This corresponds with the current investigation, revealing no immediate effects of compression socks on ankle stability. Therefore, the impact of compression socks may be more effective in fatiguing situations. The results also revealed no significant differences between the two levels of compression. Therefore, it may be reasonable to assume that there was no increased proprioceptive feedback due to localized compression grade pressure in mmHg due to the compression socks. The results of this study agree with previous research, demonstrating no effects of compression socks on enhancing ankle stability [35,36,37]. Hence, based on the current findings, both compression socks can improve postural stability when other intrinsic sensory information is absent or altered, but differences between SC and CL were not evident in acute conditions. However, these findings were attributable to all groups as no significant differences were evident between groups (CON, COP, and CAI).

Maintaining the integrity of the ankle joint is crucial for postural stability. Ankle instability could result in altered biomechanics throughout the body, making ankle stability highly correlated to the risk of injury and putting those with poor balance at greater risk of injury [9]. Lephart et al. (1998) revealed decreased sensory feedback in those with ligamentous injuries, which shows the importance of stimulation of neurological feedback mechanisms in the musculotendinous and articular structures to maintain postural stability [10]. Compression socks could improve proprioceptive feedback via localized, circumferential compression in those with CAI. Previous research has demonstrated the significance of the role of compression socks in the elderly population and individuals with CAI [15,37]. However, the current investigation revealed no significant differences among participants with no history of ankle instability, copers, and those with CAI. The rationale for the lack of significant differences between groups may be due to the testing of postural stability in mCTSIB and LOS tests on the BTrackS™ balance platform (Balance Tracking Systems, Inc., San Diego, CA, USA), as they are required to be performed in bilateral stance compared to unilateral stance. Previous literature has revealed a positive effect of compression socks on unipedal postural stability [34,38]. Furthermore, Wikstrom et al. (2010) revealed a significant difference in unipedal postural stability between individuals with CAI and healthy individuals, thus suggesting future studies to use the single-leg balance protocol on the BTrackS™ balance platform (Balance Tracking Systems, Inc., San Diego, CA, USA), especially over time with fatigue [14]. Additionally, the AJFAT questionnaire while commonly used to classify groups of healthy, coppers, and chronic ankle instability can also include other approaches such as the Cumberland Ankle Instability Tool (CAIT) to have a better classification of individuals with ankle instability.

Few limitations to this study include the testing of only bilateral stance postural stability tasks, the use of only AJFAT questionnaire to classify groups, the testing of young college-aged population, and the testing for balance only during acute conditions, without any fatigue that might further compromise postural stability, all of which should be addressed in future studies. Furthermore, there could be dynamic changes in mediolateral postural sway that may not have been accounted for with LOS alone [39], which should be addressed in future research.

## 5. Conclusions

The findings from the current study reveal that both sub-clinical and clinical-grade compression socks aid in postural stability compared to barefoot, but only when visual or somatosensory feedback is compromised, suggesting enhanced proprioceptive feedback with the compression socks. In addition, postural stability was significantly worse in sensory-challenged conditions and had significantly lower excursion limits anteriorly compared to posterior, rather than any right-left side differences, for all participants in all groups. However, significant differences were not evident between healthy controls, coppers, and chronic ankle instability individuals, which may be attributed to the type of bilateral stance balance test performed, and the classification of individuals into the groups. Therefore, compression socks may result in greater postural stability, especially when sensory feedback systems are compromised, such as in clinical populations or even in a healthy population under fatiguing conditions.

## References

[1] Horak F.B. (2006). Postural orientation and equilibrium: What do we need to know about neural control of balance to prevent falls?. Age Ageing.

[2] Nashner L.M., Shupert C.L., Horak F.B., Black F.O. (1989). Organization of posture controls: An analysis of sensory and mechanical constraints. Prog. Brain Res..

[3] Singh R.E., Iqbal K., White G. (2020). Proficiency-Based Recruitment of Muscle Synergies in a Highly Perturbed Walking Task (Slackline). Eng. Rep..

[4] Sousa A.S.P., Leite J., Costa B., Santos R. (2017). Bilateral Proprioceptive Evaluation in Individuals with Unilateral Chronic Ankle Instability. J. Athl. Train..

[5] Tuthill J.C., Azim E. (2018). Proprioception. Curr. Biol..

[6] Davids K., Shuttleworth R., Button C., Renshaw I., Glazier P. (2004). “Essential noise”—Enhancing variability of informational constraints benefits movement control: A comment on Waddington and Adams (2003). Br. J. Sports Med..

[7] Angelakos I., Mills C., O’Halloran J. (2020). The Effects of Compression Garments on Stability and Lower Limb Kinematics during a Forward Lunge. J. Hum. Kinet..

[8] Parkhurst T.M., Burnett C.N. (1994). Injury and proprioception in the lower back. J. Orthop. Sports Phys. Ther..

[9] Hasan H. (2020). Effect of Compression Socks on Postural Balance among University Netball Players. Malays. J. Mov. Health Exerc..

[10] Lephart S.M., Pincivero D.M., Rozzi S.L. (1998). Proprioception of the ankle and knee. Sports Med..

[11] Freeman M.A. (1965). Instability of the foot after injuries to the lateral ligament of the ankle. J. Bone Jt. Surg. Br..

[12] Hertel J. (2008). Sensorimotor deficits with ankle sprains and chronic ankle instability. Clin. Sports Med..

[13] Karagiannakis D.N., Iatridou K.I., Mandalidis D.G. (2020). Ankle muscles activation and postural stability with Star Excursion Balance Test in healthy individuals. Hum. Mov. Sci..

[14] Wikstrom E.A., Fournier K.A., McKeon P.O. (2010). Postural control differs between those with and without chronic ankle instability. Gait Posture.

[15] Broatch J.R., Halson S.L., Panchuk D., Bishop D.J., Waddington G. (2021). Compression enhances lower-limb somatosensation in individuals with poor somatosensation, but impairs performance in individuals wth good somatosensation. Transl. Sports Med..

[16] You S.H., Granata K.P., Bunker L.K. (2004). Effects of circumferential ankle pressure on ankle proprioception, stiffness, and postural stability: A preliminary investigation. J. Orthop. Sports Phys. Ther..

[17] Baige K., Noé F., Paillard T. (2020). Wearing compression garments differently affects monopodal postural balance in high-level athletes. Sci. Rep..

[18] Nishikawa T., Grabiner M.D. (1999). Peroneal motoneuron excitability increases immediately following application of a semirigid ankle brace. J. Orthop. Sports Phys. Ther..

[19] Hijmans J.M., Zijlstra W., Geertzen J.H.B., Hof A.L., Postema K. (2009). Foot and ankle compression improves joint position sense but not bipedal stance in older people. Gait Posture.

[20] McKeon P.O., Stein A.J., Ingersoll C.D., Hertel J. (2012). Altered plantar-receptor stimulation impairs postural control in those with chronic ankle instability. J. Sport Rehabil..

[21] Pavin L.N., Leicht A.S., Gimenes S.V., da Silva B.V.C., Simim M.A.d.M., Marocolo M., Mota G.R. (2019). Can compression stockings reduce the degree of soccer match-induced fatigue in females?. Res. Sports Med..

[22] Rozzi S.L., Lephart S.M., Sterner R., Kuligowski L. (1999). Balance training for persons with functionally unstable ankles. J. Orthop. Sports Phys. Ther..

[23] Ross S.E., Guskiewicz K.M., Gross M.T., Yu B. (2008). Assessment tools for identifying functional limitations associated with functional ankle instability. J. Athl. Train..

[24] Wikstrom E.A., Tillman M.D., Chmielewski T.L., Cauraugh J.H., Naugle K.E., Borsa P.A. (2010). Dynamic postural control but not mechanical stability differs among those with and without chronic ankle instability. Scand. J. Med. Sci. Sports.

[25] Shephard R.J. (1988). PAR-Q, Canadian Home Fitness Test and exercise screening alternatives. Sports Med..

[26] Winter D. (1995). Human balance and posture control during standing and walking. Gait Posture.

[27] Jaakkola T., Linnamo V., Woo M.T., Davids K., Piirainen J.M., Gråstén A. (2017). Effects of training on postural control and agility when wearing socks of different compression levels. Biomed. Hum. Kinet..

[28] Cohen H., Blatchly C.A., Gombash L.L. (1993). A study of the clinical test of sensory interaction and balance. Phys. Ther..

[29] Goble D.J., Baweja H.S. (2018). Normative Data for the BTrackS Balance Test of Postural Sway: Results from 16,357 Community-Dwelling Individuals Who Were 5 to 100 Years Old. Phys. Ther..

[30] Goble D.J., Brar H., Brown E.C., Marks C.R., Baweja H.S. (2019). Normative data for the Balance Tracking System modified Clinical Test of Sensory Integration and Balance protocol. Med. Devices.

[31] Goble D.J., Brown E.C., Marks C.R.C., Baweja H.S. (2020). Expanded Normative Data for the Balance Tracking System Modified Clinical Test of Sensory Integration and Balance Protocol. Med. Devices Sens..

[32] Clark S., Rose D.J., Fujimoto K. (1997). Generalizability of the limits of stability test in the evaluation of dynamic balance among older adults. Arch. Phys. Med. Rehabil..

[33] Juras G., Słomka K., Fredyk A., Sobota G., Bacik B. (2008). Evaluation of the Limits of Stability (LOS) Balance Test. J. Hum. Kinet..

[34] Chang L., Fu S., Wu S., Witchalls J., Adams R., Waddington G., Han J. (2022). Effects of graduated compression socks on ankle inversion proprioception of half-marathon runners at different running distances. J. Sci. Med. Sport.

[35] Bendahou M., Khiami F., Saïdi K., Blanchard C., Scepi M., Riou B., Besch S., Hausfater P. (2014). Compression stockings in ankle sprain: A multicenter randomized study. Am. J. Emerg. Med..

[36] Woo M.T., Davids K., Liukkonen J., Jaakkola T., Chow J.Y. (2014). Effects of Textured Compression Socks on Postural Control in Physically Active Elderly Individuals. Procedia Eng..

[37] Woo M.T., Davids K., Liukkonen J., Chow J.Y., Jaakkola T. (2018). Immediate effects of wearing knee length socks differing in compression level on postural regulation in community-dwelling, healthy, elderly men and women. Gait Posture.

[38] Michael J.S., Dogramaci S.N., Steel K.A., Graham K.S. (2014). What is the effect of compression garments on a balance task in female athletes?. Gait Posture.

[39] Watson F., Fino P.C., Thornton M., Heracleous C., Loureiro R., Leong J.J.H. (2021). Use of the margin of stability to quantify stability in pathologic gait—A qualitative systematic review. BMC Musculoskelet. Disord..

